# Association between age-related cataract and blepharoptosis in Korean adults: a population-based study

**DOI:** 10.1038/s41598-021-04381-7

**Published:** 2022-01-10

**Authors:** Kyung-Sun Na, Su-Kyung Jung, Younhea Jung, Kyungdo Han, Jiyoung Lee, Ji-Sun Paik, Suk-Woo Yang

**Affiliations:** 1 Department of Ophthalmology, Yeouido St. Mary’s Hospital, College of Medicine, The Catholic University of Korea, 10, 63-ro, Yeongdeungpo-gu, Seoul, 07345 Republic of Korea; 2 Eyeclinic, Center for Clinical Center, National Cancer Center, Goyang-si, South Korea; 3 Department of Statistics and Actuarial Science, Soongsil University, Seoul, Republic of Korea; 4 Department of Ophthalmology, Seoul St. Mary’s Hospital, College of Medicine, The Catholic University of Korea, Seoul, Republic of Korea

**Keywords:** Diseases, Ageing

## Abstract

Cataract and blepharoptosis are both commonly encountered ophthalmic problems in older adults. Since they share similar risk factors, it is plausible that there may be an association between the two conditions. We examined data from the Korean National Health and Nutrition Examination Survey (KNHANES) 2010–2012 to determine if there is an association between age-related cataract and blepharoptosis. Multivariable adjusted logistic regression analysis was conducted to examine the odds ratio (OR) and 95% confidence interval (CI) for association of each specific type of cataract with presence of blepharoptosis. Of the 10,387 eligible participants, 4782 (46.0%) had cataract and 1419 (15.8%) had blepharoptosis. There were more participants with blepharoptosis in the cataract group, compared with those in the no cataract group. After adjusting for potential confounders, participants with blepharoptosis had a higher risk of total cataract (OR: 1.557, 95% CI 1.201–2.019) and nuclear subtype cataract (OR: 1.305, 95% CI 1.050–1.620). Blepharoptosis was associated with significantly higher odds of cataract in obese participants when compared with non-obese participants (p for interaction = 0.0236). Our study revealed a positive association between age-related cataract and blepharoptosis; it suggests that thorough ophthalmic assessment is needed when assessing patients who are planning cataract or blepharoptosis surgery.

## Introduction

Age-related cataract is a multifactorial disease which is a major cause of visual impairment and vision loss worldwide^1^. It is expected that age-related cataracts will continue to be an important global health issue due to increasing life expectancy^1,2^. Although the pathogenesis of cataract is not completely defined, oxidative stress from the aging process has a major undisputed role in lens opacity^3,4^. Risk factors are known to include age, diabetes, smoking, ultraviolet radiation, metabolic diseases, obesity, and genetic influence^1,5–7^. Different risk factors seem to play a role for different subtypes of cataract, including cortical, nuclear, anterior polar, and posterior subcapsular cataract^8,9^. Another common age-related ophthalmic problem is blepharoptosis, which is defined as an abnormal, low-lying eyelid margin with the eye in primary gaze^10,11^. Epidemiologic studies have reported that the prevalence of blepharoptosis is over 10% in middle aged to older adults^11,12^. It develops from a degenerative process involving the levator aponeurosis as a result of aging combined with periorbital changes. Although the risk factors of blepharoptosis are not clearly defined, aging, obesity, hypertension, diabetes, and possibly smoking are reported to increase the prevalence of blepharoptosis^11,13^.

Given the fact that cataract and blepharoptosis are both commonly encountered ophthalmic problems in older adults, and share similar risk factors, it is plausible that cataract and blepharoptosis are associated in their prevalence. Until now, there are no previous studies on the association between cataract and blepharoptosis, although postoperative blepharoptosis is known to be acquired after intraocular surgery such as cataract procedures. To test our hypothesis, we examined the association between the two ophthalmic conditions using a large national patient database. The Korea National Health and Nutrition Examination Survey (KNHANES) is a nationally representative survey conducted by the Korean Ministry of Health and Welfare that accumulates data on citizens including vision status, healthcare use, and other socio-demographic factors^14^. Commencing in the latter half of 2008, ophthalmologic examinations were included in the survey in order to investigate the prevalence and risk factors of common eye diseases^15^. KNHANES results and statistics are readily available at http://knhanes.cdc.go.kr. Our goal was to use ophthalmologic examination results from KNHANES to evaluate the association between cataract and blepharoptosis.

## Results

### Demographics of general characteristics of the study population

**Figure 1:**
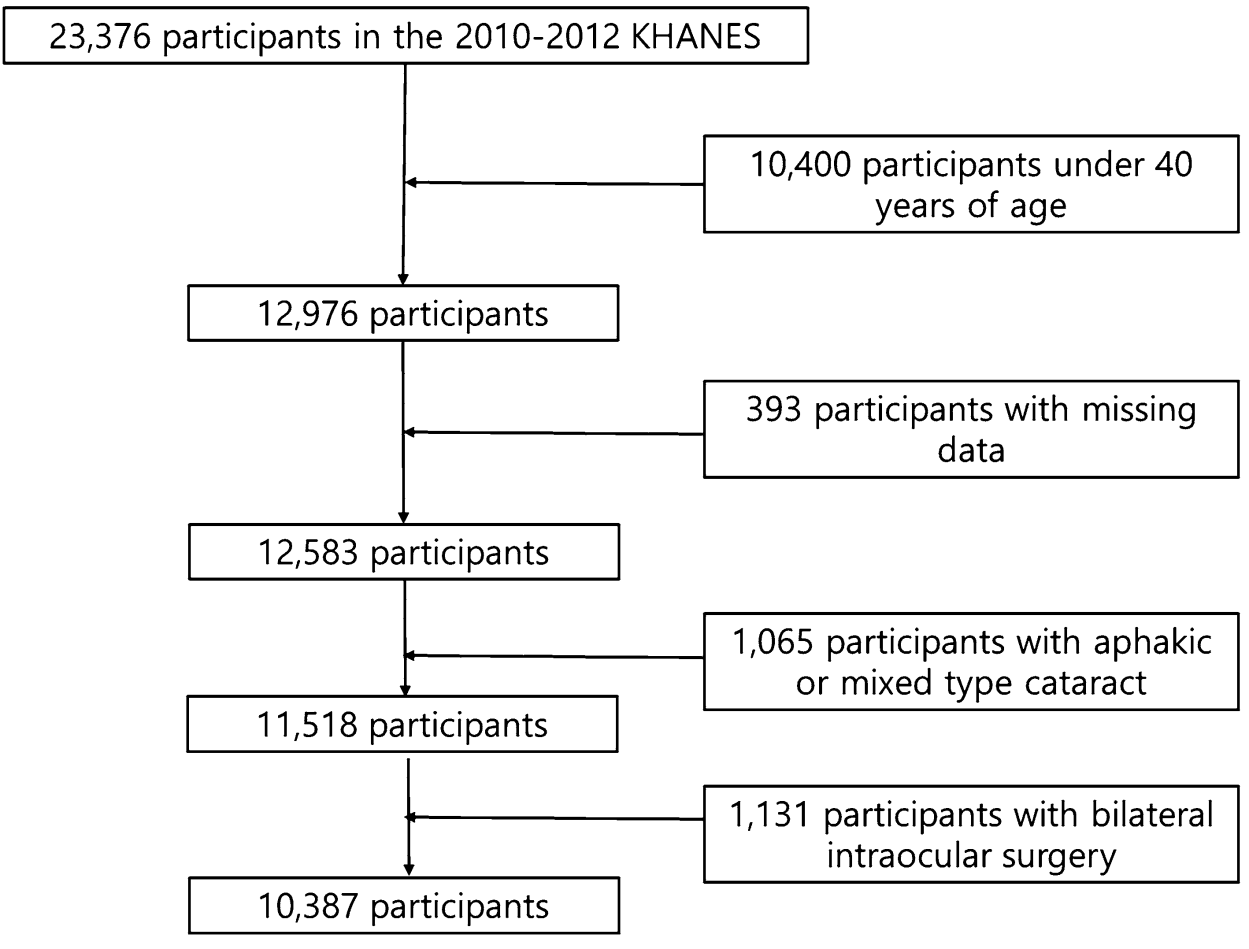
Flow chart of subjects.

There were 23,376 participants in the 2010–2012 KNHANES. We excluded 10,400 individuals younger than 40 years of age, 393 subjects with missing data of cataract or blepharoptosis, 1065 subjects with aphakic or mixed type cataract, and 1131 subjects with bilateral intraocular surgeries (Fig. 1). Table 1 shows the demographic and clinical characteristics of the study population. Of the remaining 10,387 participants, 4782 subjects (46.0%) had cataract and 1419 subjects (15.8%) had blepharoptosis. Most potential confounders were significantly different (p < 0.05) between the groups with cataract and without cataract except for sex and BMI. Similarly, most potential confounders were significantly different (p < 0.05) between groups with blepharoptosis and without blepharoptosis except for sex, current smoking, and stress perception.

**Table 1:** General characteristics of study participants.

Number	Cataract			Number	Blepharoptosis		
	No	Yes	p value		No	Yes	p value
	5605	4782			8968	1419	
Age	49.57 ± 0.17	62.53 ± 0.31	< 0.0001	Age	53.45 ± 0.18	62.6 ± 0.44	< 0.0001
**Sex**			0.8438	**Sex**			0.173
Male	48.7 (0.66)	48.48 (0.79)		Male	48.36 (0.51)	50.53 (1.43)	
Female	51.3 (0.66)	51.52 (0.79)		Female	51.64 (0.51)	49.47 (1.43)	
**Education**			< 0.0001	**Education**			< 0.0001
Elementary school	16.04 (0.77)	47.18 (1.25)		Elementary school	24.91 (0.73)	50.78 (2.02)	
Middle school	15.3 (0.64)	16.29 (0.75)		Middle school	15.6 (0.54)	16.27 (1.26)	
High school	40.21 (0.94)	24.47 (0.98)		High school	36.04 (0.77)	20.33 (1.42)	
University	28.44 (1.04)	12.06 (0.83)		University	23.45 (0.86)	12.62 (1.29)	
**Household income**			< 0.0001	**Household income**			< 0.0001
Lower	11.04 (0.64)	31.59 (1.05)		Lower	17.12 (0.63)	32.27 (1.76)	
Middle lower	26.94 (0.96)	26.43 (0.98)		Middle lower	26.5 (0.78)	28.6 (1.76)	
Middle upper	29.16 (0.91)	21.04 (0.96)		Middle upper	26.8 (0.74)	20.5 (1.42)	
Upper	32.85 (1.1)	20.94 (1)		Upper	29.59 (0.89)	18.63 (1.57)	
Current smoking	24.56 (0.79)	19.86 (0.82)	0.0001	Current smoking	22.73 (0.6)	22.94 (1.49)	0.8977
Drink (month)	58.88 (0.81)	45.71 (0.96)	< 0.0001	Drink (month)	54.79 (0.66)	46.72 (1.67)	< 0.0001
Regular exercise	21.21 (0.72)	17.66 (0.94)	0.0017	Regular exercise	20.33 (0.62)	16.33 (1.49)	0.0141
Place (urban)	78.63 (1.96)	71.26 (2.6)	0.0008	Place (urban)	76.66 (1.94)	69.42 (3.29)	0.0064
Spouse	87.74 (0.58)	77.7 (0.85)	< 0.0001	Spouse	85.12 (0.52)	74.75 (1.45)	< 0.0001
DM	8.3 (0.52)	17.94 (0.7)	< 0.0001	DM	10.82 (0.42)	20.67 (1.31)	< 0.0001
Hypertension	29.58 (0.83)	51.2 (1.02)	< 0.0001	Hypertension	35.63 (0.7)	54.56 (1.81)	< 0.0001
Stress perception	25.23 (0.71)	22.82 (0.76)	0.0189	Stress perception	24.28 (0.56)	24.51(1.43)	0.8765
Sun exposure	13 (0.85)	19.75 (1.48)	< 0.0001	Sun exposure	15.07 (0.91)	19.42 (1.96)	0.0137
BMI	24.12 ± 0.05	24.08 ± 0.07	0.6719	BMI	24.06 ± 0.04	24.44 ± 0.1	0.0003
WC	82.2 ± 0.17	83.91 ± 0.2	< 0.0001	WC	82.55 ± 0.14	85.16 ± 0.31	< 0.0001
Vit D level	17.83 ± 0.16	18.67 ± 0.21	< 0.0001	Vit D level	18.09 ± 0.15	18.59 ± 0.26	0.0494

Geometric means/Values are presented as measn (SD) for continuous variable and n (%) for categorical variables.

*DM* diabetes mellitus, *BMI* body mass index, *WC* waist circumference, *Vit D* vitamin D.

### The prevalence of cataract according to MRD1 and levator function level

Table 2 shows the prevalence of cataract according to MRD1 and levator function level. Assuming an MRD1 < 2 mm equates to blepharoptosis, there were more participants with blepharoptosis in the cataract group, compared with participants in the no cataract group. Similarly, there were more participants with LFT < 7 mm in the cataract group, compared with people in the no cataract group. All of these results were statistically significant (< 0.001).

**Table 2:** Cataract prevalence according to MRD1 and levetor function level.

Number	Cataract		p-value
	No 5605	Yes 4782	
**MRD1**^**a**^** right (mm)**			< 0.0001
≥ 4	41.71 (1.65)	22.36 (1.43)	
3–3.9	35.44 (1.26)	33.61 (1.22)	
2–2.9	17.27 (1.27)	25.38 (1.08)	
1–1.9	4.45 (0.45)	13.78 (0.84)	
< 1	1.14 (0.22)	4.87 (0.48)	
**MRD1 left (mm)**			< 0.0001
≥ 4	41.76 (1.65)	22.64 (1.47)	
3–3.9	35.31 (1.25)	33.16 (1.21)	
2–2.9	16.99 (1.25)	25.77 (1.11)	
1–1.9	5.02 (0.49)	13.48 (0.81)	
< 1	0.92 (0.18)	4.95 (0.51)	
**Levator function (mm)**			< 0.0001
≥ 12	70.71 (1.5)	47.73 (1.57)	
8–11	27.42 (1.44)	45.08 (1.48)	
5–7	1.68 (0.25)	6.22 (0.61)	
≤ 4	0.19 (0.07)	0.96 (0.28)	
**Levator function (mm)**			< 0.0001
≥ 12	71.12 (1.48)	48.57 (1.55)	
8–11	26.91 (1.43)	44.16 (1.42)	
5–7	1.78 (0.27)	6.34 (0.66)	
≤ 4	0.19 (0.07)	0.93 (0.28)	

^a^Marginal reflex distance 1 (MRD 1) was measured from the central upper lid margin to the pupillary light reflex on the cornea.

### Relation between blepharoptosis and cataract subtypes and the OR for cataract according to MRD1

**Figure 2:**
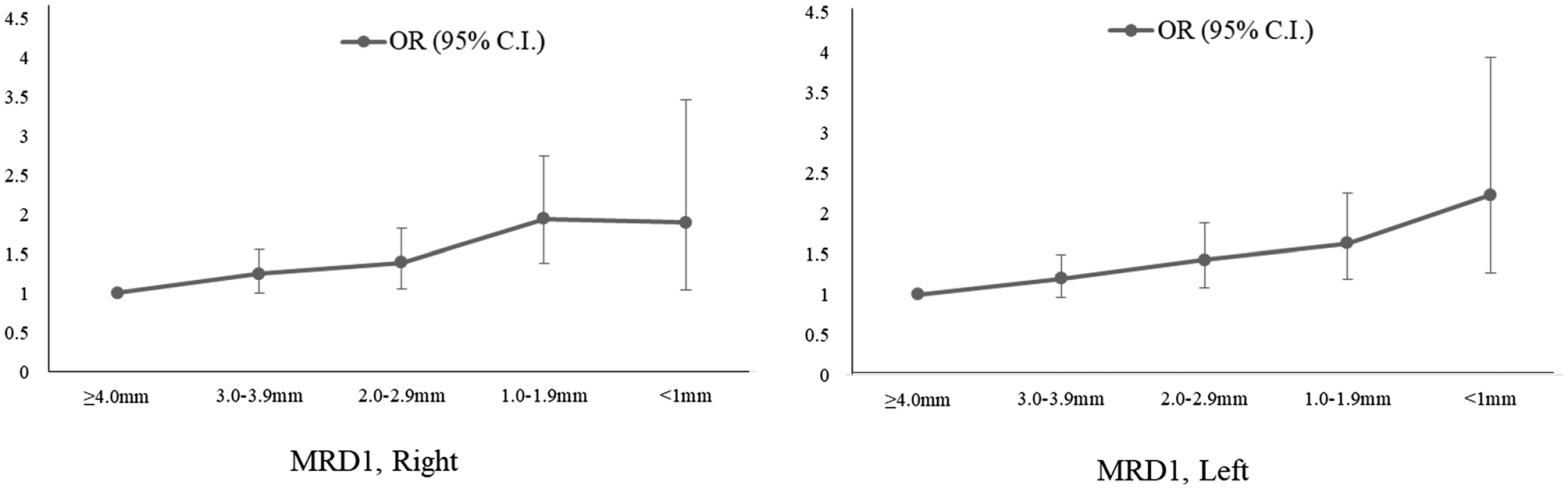
OR with 95% CI for cataract incidence according to MRD1 level in both eyes. As the level of MRD1 decreased, there is a tendency to increase odds ratio of cataract in the right eye and the left eyes and it is statistically significant (p = 0.0038, right eye, p = 0.0118, left eye, respectively).

Table 3 shows the OR of different subtypes of cataract in the participants with blepharoptosis in the multivariable logistic regression models. After adjusting for potential confounders (model 3, age, sex, smoking, drink, exercise, BMI, income, education level, vit D, and sun exposure), subjects with blepharoptosis have a higher risk of total cataract (OR: 1.557, 95% CI 1.201–2.019) and nuclear subtype cataract (OR: 1.305, 95% CI 1.050–1.620). Figure 2 shows that, as the level of MRD1 decreased, there was a tendency for the OR of cataract in the right eye and the left eye to increase (p = 0.0038, right eye, p = 0.0118, left eye, respectively).

**Table 3:** ORs (95% CI) of the different subtypes of cataract for the subjects with blepharoptosis.

Dependent variable	Blepharoptosis	% (SE)	OR (95% CI)		
			Model 1	Model 2	Model 3
Cataract	No	34.53 (1.12)	1	1	1
	Yes	66.23 (2.18)	3.717 (3.076, 4.493)	1.578 (1.217, 2.047)	1.557 (1.201, 2.019)
Cortical	No	8.05 (0.62)	1	1	1
	Yes	12.76 (1.35)	1.671 (1.301, 2.148)	1.026 (0.792, 1.329)	1.006 (0.778, 1.301)
Nuclear	No	21.34 (1.03)	1	1	1
	Yes	40.12 (2.22)	2.47 (2.065, 2.956)	1.322 (1.063, 1.644)	1.305 (1.050, 1.620)
APC	No	0.86 (0.15)	1	1	1
	Yes	1.39 ( (0.37)	1.637 (0.938, 2.857)	1.043 (0.584, 1.863)	1.024 (0.568, 1.844)
PSC	No	0.23 (0.06)	1	1	1
	Yes	0.74 (0.25)	3.303 (1.511, 7.218)	1.835 (0.674,4.995)	1.768 ( (0.661, 4.731)

Model 1: unadjusted, Model 2: adjusted for sex and age, Model 3: adjusted for sex, age, smoking, drink, regular exercise, BMI, income, education level, vitamin D, and sun exposure.

*APC* anterior polar cataract, *PSC* posterior subcapsular opacity.

### Subgroup analyses of cataract prevalence according to with/without blepharoptosis

**Figure 3:**
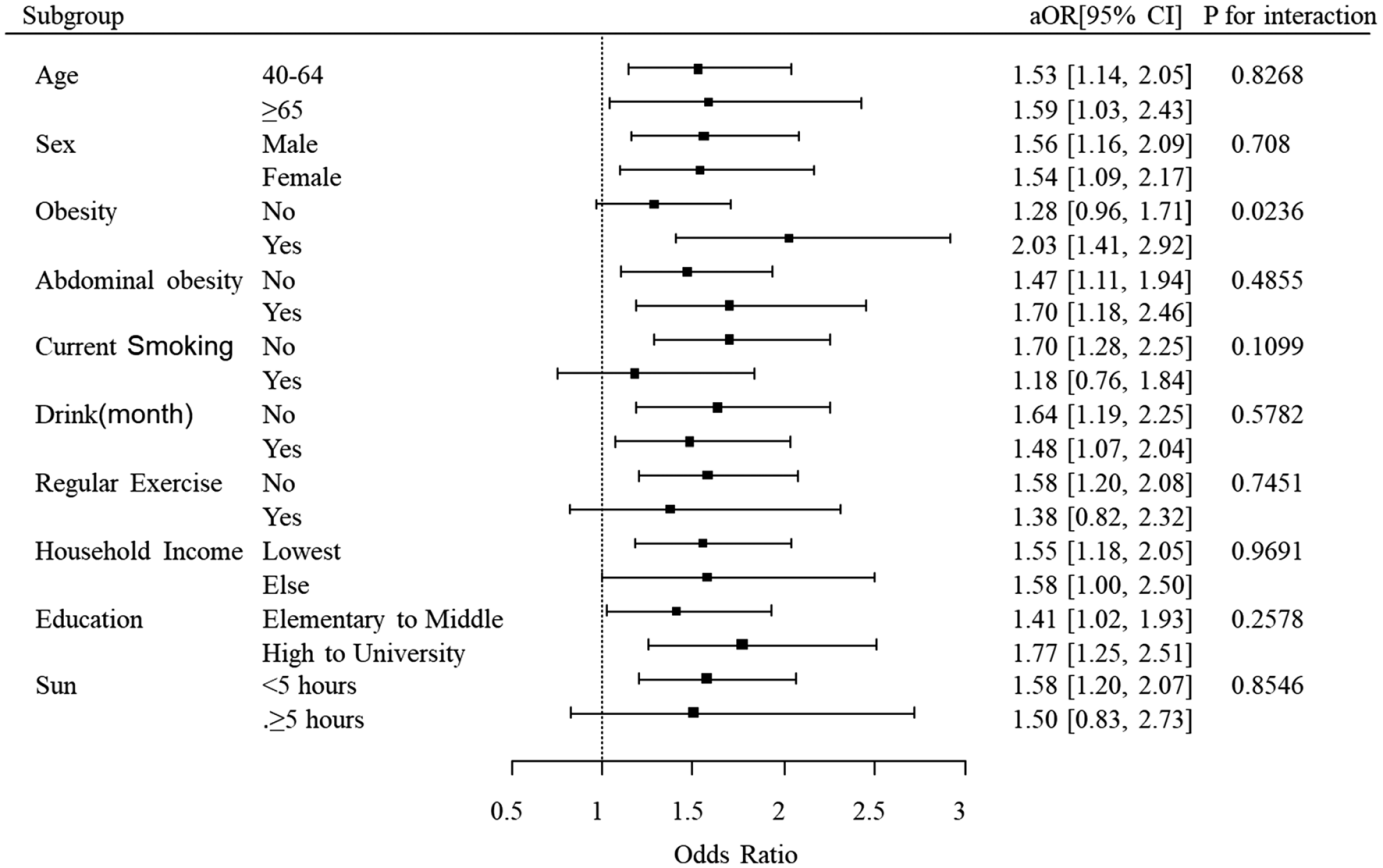
Association between cataract and blepharoptosis according to subgroup. There was a significant heterogeneity in the odds ratios for cataract associated with blepharoptosis in the obesity and non-obesity (p for interaction 0.0236) after adjusted for age, sex, smoking, drinking, regular exercise, BMI, regular income, education level, and vitamin D level.

Figure 3 shows the results of subgroup analyses of cataract and blepharoptosis prevalence according to age, sex, obesity, abdominal obesity, current smoking, drink, regular exercise, household income, education, and sun exposure. In the subgroup analyses, there was a significant heterogeneity in the ORs for cataract associated with blepharoptosis in cases of obesity and non-obesity. Blepharoptosis is associated with significantly higher odds of cataract in obese participants (OR: 2.03, 95% CI 1.41–2.92) when compared with non-obese participants (OR: 1.28, 95% CI 0.96–1.71) [p for interaction = 0.0236]. In other subgroup analyses, there were no significant differences in the association between cataract and blepharoptosis.

## Discussion

In this nationwide representative study based on a stratified, multistage, probability-sampling design, we found that there was a positive association between cataract and blepharoptosis prevalence in participants over 40 years old. Participants with blepharoptosis had a higher risk of any cataract and nuclear subtype after adjusting for confounding factors. Among the participants, 46.0% had cataract and 15.8% had blepharoptosis, which was similar to previous epidemiologic studies in Asia^11,16–18^. Most subgroups did not show any significant interactions with cataract and blepharoptosis except for obesity. Obese participants with blepharoptosis showed greater odds of cataract compared with non-obese participants.

Previous studies have reported that risk factors for cataract are age, diabetes, obesity, metabolic syndrome, smoking, educational status, sunlight exposure and estrogen replacement therapy^5–9^. However, little is known of the prevalence and risk factors for each subtypes of cataract^5,8,19^. Given the fact that the prevalence of subtypes of cataract varies according to race and environment^20,21^, this epidemiologic study of mono-ethnicity under homogenous environment (climates, food culture) contributes to defining the pathophysiology of cataract. We found that any cataract is positively associated with blepharoptosis after all confounding variables were adjusted. And among all subtypes, only nuclear cataract was shown to be significantly associated with blepharoptosis. Nuclear sclerosis of the lens may be a marker for changes in structural proteins of the whole body, mainly resulting from oxidation that cause diminishes function^3,4,22^. Previous studies revealed that nuclear cataract appeared to be associated with smoking and metabolic syndrome, whereas the other subtypes did not^5^. Chronic oxidative stress with aging may be a link between cataract and blepharoptosis development^22,23^. Oxidative stress is a well-known initiating factor for various ophthalmic disorders including cataract, glaucoma and macular degeneration^3^. Although mechanisms underlying the pathologic study of the levator aponeurosis remain unknown, there is some evidence that oxidative stress also affects blepharoptosis development^23^. Histopathologic examination of the levator muscle showed that the expression of 8-hydroxydeoxyguanosine, a marker of oxidative stress, was higher in the levator aponeurosis tissues of blepharoptosis compared with normal tissue^23^. These results indicate that oxidative DNA damage of striated muscle contributes to muscle dysfunction and muscle contraction disorders in blepharoptosis patients.

Interestingly, our study results showed that obese individuals with blepharoptosis showed significantly greater odds of cataract. These findings suggest that there is a clinical implication of obesity with regard to the risk of blepharoptosis and cataract. Additionally, findings from the subgroup analysis revealed a significant interaction exists between blepharoptosis and cataract regarding obesity. High BMI has been reported to be a risk factor for both blepharoptosis and cataract^7,13,24^. In previous studies, obesity may be considered as a possible determinant of age-related blepharoptosis, suggested by histologic evidence of fatty infiltration of the upper-eyelid elevators or in the status of the aponeurosis^25^. Also, obesity is associated with insulin resistance on peripheral glucose and fatty acid utilization, often leading to type 2 diabetes mellitus, dyslipidemia, hypertension, and cardiovascular diseases^26^. Metabolic syndrome is a known risk factor for cataract^27^, and taken together, obesity with metabolic syndrome would be a connecting link in the association of blepharoptosis and cataract.

Blepharoptosis is known to be a complication after cataract surgery^28^. In modern clear corneal suture-less phacoemulsification cataract surgery, it has been reported that there is more than 3% of cases with postoperative persistent clinically significant blepharoptosis^29^. In our study, we excluded participants who had history of intraocular surgery, but those who underwent unilateral cataract surgery were included in the analysis; therefore, this study design could not distinguish between cataract in the right and left eye, and it is possible that the association between blepharoptosis and cataract was not analyzed in the same eye. However, we found that only 2.7% of those with blepharoptosis had it unilaterally, which would not affect the results of the current study (Supplement Table 1). Additionally, the MRD1 difference was not statistically significant between the right and left eyes in all subjects (Supplement Table 2). In cataract patients, it is possible that levator function is low preoperatively even though clinically significant blepharoptosis is not apparent. Surgeons should be aware that cataract and blepharoptosis may coexist especially if the patient is obese. With a MRD1 of 2 mm, 25% of superior visual field defect is known to be disturbed in various studies^30,31^. In addition, ptosis and upper eyelid blepharoplasty surgery were found to be functionally beneficial for patients with MRD1 of 2 mm or less in the primary gaze. In this context, physicians may neglect cataract if the individual has severe blepharoptosis, because blepharoptosis per se would lead to visual impairment. Conversely, it is applied to the patient complaining of cataract in whom not only cataract affects the vision but also co-existing blepharoptosis.

There are some limitations in this study. First, this study is based on retrospective self-reported data; therefore, there may be a recall bias in analyzing socioeconomic information. We could not consider medication use, including glucocorticoids, nonsteroidal anti-inflammatory drugs, thyroid hormone, and multivitamins which might affect the cataract development. Second, it is not possible to deduce any causal relationship between cataract and blepharoptosis from the cross-sectional design of the study. Lastly, we included subjects with unilateral cataract surgery. There may be a small number of subjects who showed blepharoptosis after cataract surgery. However, the statistical benefit of maintaining a large number of subjects without excluding bilateral cataract surgery exceeds the risk of bias. Despite these limitations, the main strength of our study is that it is the first study to confirm the association between cataract and blepharoptosis, suggesting the clinical implications of cataract in assessing blepharoptosis, and vice versa. Furthermore, the data used was from a large scale nationwide database that represents the Korean population; thus, it might provide epidemiologic evidence for single ethnicity. In KNHANES, the KCDCP and the Korean Ophthalmological Society conducted team education, and the ophthalmologists participating in this survey were required to complete a training course and undergo supervised practice before working in the actual survey field^15^. Thus, the cataract diagnosis using slit lamp examination and blepharoptosis determined by measuring the MRD1 were both reliably assessed. Additionally, this study accounted for a comprehensive number of potential confounding factors which might be related to both cataract and blepharoptosis.

In conclusion, our study found positive associations between cataract and blepharoptosis after adjusting for possible confounding factors. Especially, the nuclear cataract showed a significant association with blepharoptosis prevalence. Our study suggests that thorough ophthalmic assessment including eyelid and lens is needed when assessing patients who are planning cataract or blepharoptosis surgery. Further studies such as cohort studies are warranted to confirm these associations between cataract and blepharoptosis.

## Methods

### Study population and data collection

Data from the Korean National Health and Nutrition Examination Survey (KNHANES) 2010–2012 were analyzed in the present study. The KNHANES is performed annually to monitor the general health and nutritional status of the South Korean population by the Korean Centres for Disease Control and Prevention (KCDC) and the Korean Ministry of Health and Welfare. This study was reviewed and approved by the Institutional Review Board/Ethics Committee of the Catholic University of Korea in accordance with the Declaration of Helsinki (IRB number: SC20ZISE0140), and all participants provided written informed consent prior to participation.

### The assessment of blepharoptosis

Blepharoptosis was defined as the presentation of a marginal reflex distance 1 (MRD1) of < 2 mm (reference). The MRD1 was measured from the central upper lid margin to the pupillary light reflex on the cornea. The values of MRD1 were measured and categorized into five groups (1) ≥ 4 mm (millimeters); (2) 3.0–3.9 mm; (3) 2.0–2.9 mm; (4) 1.0–1.9 mm; and (5) < 1 mm in either eye. The levator function (LFT, levator function test) was estimated by measuring the upper eyelid excursion from down-gaze to up-gaze eliminating the frontalis muscle function, and categorized into four subgroups (1) ≤ 12 mm (excellent); (2) 8–11 mm (good); (3) 5–7 mm (fair); and (4) < 4 mm (poor). All participants’ lid positions were examined by specially trained examiners who had been working as ophthalmologic residents for over 3 years and this was considered as one of the routine ophthalmologic measures.

### The assessment cataract formation

Lens opacity was diagnosed by trained ophthalmologic residents by using the Lens Opacity Classification System (LOCS) II system^32^. The Lens Opacities Classification System II (LOCS II) was used to classify opacities until into seven cortical (C0, Ctr, CI, CII, CIII, CIV, CV), five nuclear (NO, NI, NII, NIII, NIV), and five PSC (P0, PI, PII, PIII, PIV) grades of increasing severity, according to photographic standards. Subjects with aphakia or pseudophakia were also documented even though they were excluded in the present study. The severity or grade of lens opacity was not recorded, and only the subtype of cataract present, such as nuclear, cortical, posterior subcapsular, and anterior polar cataracts, was recorded. The criteria of lens opacities used in the present study were similar to those used in the previous reports^33,34^. Nuclear, cortical, posterior subcapsular cataracts, and anterior polar cataracts were noted in individuals with the same single type of opacity present in both eyes. They were decided in individuals with only a single type opacity with a LOCS II ≥ 2, presented between both eyes. If a participant had unilateral lens extraction, the contralateral phakic eye was used to define the lens opacity type in that individual. Subjects with mixed-type opacities and subjects who had bilateral lens extraction were excluded. The quality of the ophthalmologic examinations was verified by the Epidemiologic Survey Committee of the Korean Ophthalmologic Society.

### Other variables

Questionnaires were used to collect demographic information, smoking history, alcohol consumption, regular exercise, place to live, presence of spouse, presence of mental stress, educational level, general income, and usual sun exposure. Current smokers were defined as participants who currently smoked and had smoked more than 100 cigarettes in their lifetime. Alcohol consumers were defined as participants who drank more than once a month within the last year. Participants who performed moderate exercise at least five times per week for 30 min or more per session, or who performed vigorous exercise at least three times per week for 20 min or more per session, were considered regular exercisers. Stress perception was defined as the percentage which participants felt more stressful during daily life. Participants with sun exposure were defined as those with more than 5 h of sun exposure. All anthropometric measurements were obtained by a specially trained examiner. Body mass index (BMI) was obtained as the participant’s weight in kilograms divided by the square of the participant’s height in meters. Waist circumstance was measured in a horizontal plane at the level of the midpoint between the iliac crest and the costal margin. Systemic hypertension was defined as measured systolic blood pressure ≥ 140 mmHg and/or diastolic blood pressure ≥ 90 mmHg or if the patients were currently using systemic antihypertensive medication. Blood pressure was examined in a seated position and after at least 5 min of rest. Diabetes was defined as a fasting blood glucose ≥ 126 mg/dL or if the patient was currently using systemic antidiabetic medication or scheduled insulin injection. Blood glucose levels were obtained after fasting for a minimum of 8 h.

### Statistical analyses

The data were expressed as numbers and percentage (categorical) or mean ± standard error (continuous). Differences in the distribution of continuous and categorical variables by cataract were evaluated using t-tests or the χ^2^ test, respectively. Multivariable adjusted logistic regression analysis was conducted to examine the odds ratio (OR) and 95% confidence interval (CI) for association of each specific type of cataract with presence of blepharoptosis. Model 1 was unadjusted, model 2 was only adjusted by age and sex, and model 3 was additionally adjusted by smoking, drinking, regular exercise, BMI, general income, education level, vitamin D (Vit D), and sun exposure, in addition to age and sex. Subgroup analysis was adjusted for age, sex, smoking, drinking, regular exercise, BMI, general income, education level, and Vit D level. Statistical analyses were performed using SAS 9.4 version software (SAS Institute, Cary, NC, USA) to account for the complex sampling design and provide national prevalence estimates. A p-value less than 0.05 was considered statistically significant.

## Supplementary Information

Supplementary Tables.
