# Supplementary material for: Association between age-related cataract and blepharoptosis in Korean adults: a population-based study
Source: Sci Rep. 2022 Jan 10;12:389. doi: 10.1038/s41598-021-04381-7 (PMC8748712; doi:10.1038/s41598-021-04381-7)
Supplement: Supplementary file 1 — Supplementary Tables. [file 41598_2021_4381_MOESM1_ESM.docx]

Supplement Table 1. The MRD1 levels of upper lids of both eyes for total participants.

| MRD1 of left upper lid | MRD1 of right upper lid | N (%) |
| --- | --- | --- |
| ≥ 2.0 mm | ≥ 2.0 mm | 8,968 (86.34) |
|  | < 2.0 mm | 132 (1.27) |
| < 2.0 mm | ≥ 2.0 mm | 148 (1.42) |
|  | < 2.0 mm | 1,139 (10.97) |

Supplement Table 2. The participants’ distribution according to MRD1 level.

| MRD1 level | Right /weighted % (SE) | Left  /weighted % (SE) | P value |
| --- | --- | --- | --- |
| ≥ 4.0 mm | 34.44 (1.33) | 34.31 (1.31) | 0.2491 |
| 3.0-3.9 mm | 34.49 (0.99) | 34.74 (1.0) |  |
| 2.0-2.9 mm | 20.35 (0.95) | 20.37 (0.95) |  |
| 1.0-1.9 mm | 8.25 (0.48) | 8.02 (0.48) |  |
| < 1.0 mm | 2.46 (0.25) | 2.57 (0.25) |  |
